# Ultra-long-acting removable drug delivery system for HIV treatment and prevention

**DOI:** 10.1038/s41467-018-06490-w

**Published:** 2018-10-08

**Authors:** Martina Kovarova, S. Rahima Benhabbour, Ivana Massud, Rae Ann Spagnuolo, Brianna Skinner, Caroline E. Baker, Craig Sykes, Katie R. Mollan, Angela D. M. Kashuba, J. Gerardo García-Lerma, Russell J. Mumper, J. Victor Garcia

**Affiliations:** 1 Division of Infectious Diseases, Center for AIDS Research, Department of Medicine, School of Medicine, University of North Carolina at Chapel Hill, 120 Mason Farm Rd. CB 7042, Chapel Hill, NC 27599 USA; 2 Division of Molecular Pharmaceutics, UNC Eshelman School of Pharmacy, University of North Carolina at Chapel Hill, 125 Mason Farm Rd. 4205, Chapel Hill, NC 27599 USA; 3 Laboratory Branch, Division of HIV/AIDS Prevention, National Center for HIV/AIDS, Viral Hepatitis, STD, and TB Prevention, Centers for Disease Control and Prevention, 1600 Clifton Rd, MS G45, Atlanta, GA 30329 USA; 4 Comparative Medicine Branch, Division of Scientific Resources, National Center for Emerging and Zoonotic Infectious Diseases, Centers for Disease Control and Prevention, Atlanta, GA 30329 USA; 5 Division of Pharmacotherapy and Experimental Therapeutics, UNC Eshelman School of Pharmacy, University of North Carolina at Chapel Hill, 120 Mason Farm Rd. CB#7361, Chapel Hill, NC 27599 USA; 6 The University of North Carolina Center for AIDS Research, 3126 McGavran-Greenberg Hall, Chapel Hill, NC 27599 USA

## Abstract

Non-adherence to medication is an important health care problem, especially in the treatment of chronic conditions. Injectable long-acting (LA) formulations of antiretrovirals (ARVs) represent a viable alternative to improve adherence to HIV/AIDS treatment and prevention. However, the LA-ARV formulations currently in clinical trials cannot be removed after administration even if adverse events occur. Here we show an ultra-LA removable system that delivers drug for up to 9 months and can be safely removed to stop drug delivery. We use two pre-clinical models for HIV transmission and treatment, non-human primates (NHP) and humanized BLT (bone marrow/liver/thymus) mice and show a single dose of subcutaneously administered ultra-LA dolutegravir effectively delivers the drug in both models and show suppression of viremia and protection from multiple high-dose vaginal HIV challenges in BLT mice. This approach represents a potentially effective strategy for the ultra-LA drug delivery with multiple possible therapeutic applications.

## Abstract (web summary)

Long-acting (LA) formulations of antiretroviral (ARV) drugs are an alternative approach to improve adherence for HIV treatment and prevention. Here the authors show a removable biodegradable ultra-LA-ARV drug system that effectively delivers drug, controls viremia and prevents infection in animal models of HIV infection.

## Introduction

Adherence to medication is essential to treatment success^1^. In most cases the extent to which patients are able to follow prescribed treatments determines the final outcome. This is particularly important in the treatment of chronic conditions like mental illnesses, hypertension, diabetes, and HIV/AIDS^1^. Sustained drug release has successfully improved adherence in patients with schizophrenia^2^ and as contraceptives^3^. Long-acting (LA) injectable formulations of ARV can increase adherence and effectiveness of HIV treatment and prevention^4^. In particular, LA formulations can (1) simplify dosing schedules, (2) reduce possible side effects, (3) provide constant concentration of drug, and (4) have a positive effect on patient’s overall quality of life. LA-ARV formulations currently in clinical trials, are formulated as nanosuspensions for injections every 8 weeks^5–7^ and it is impossible to remove the injected nanosuspension from the body in the case of a medical emergency.

To address this significant limitation and to extend the duration of treatment, an ultra-LA injectable and removable formulation for HIV PrEP based on in situ forming implant technology was developed. This allows drug administration by subcutaneous injection followed by implant solidification in vivo and subsequent biodegradation of the implant resulting in sustained drug release^8,9^. The efficacy of drug delivery via ultra-LA formulation in the context of HIV is evaluated in BLT humanized mice, systemically reconstituted with human hematopoietic cells^10^. BLT mice have been extensively used for HIV transmission, replication, and persistence studies^11–14^. Importantly, humanized BLT mice allow evaluation of HIV treatment and prevention strategies with a variety of transmitted/founder HIV-1 isolates via relevant routes of transmission.

## Results

### Preparation of ultra-LA dolutegravir

**Fig. 1:**
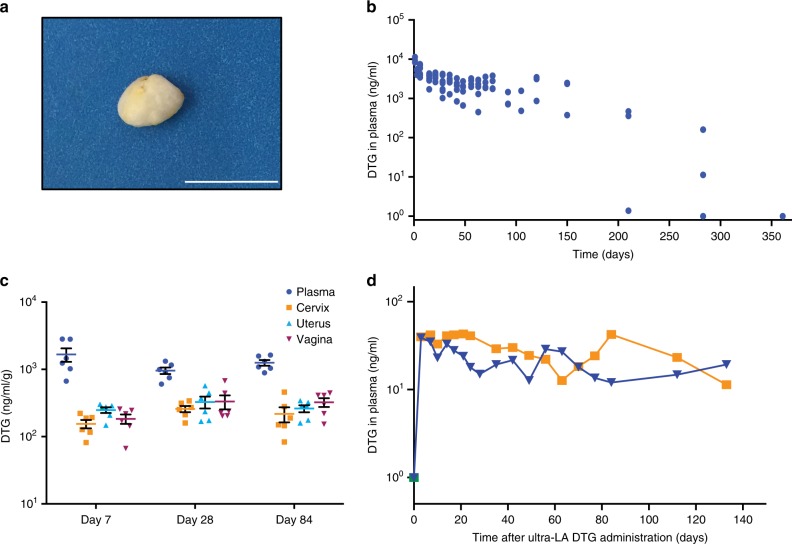
Implant formation and dolutegravir concentration in plasma and female reproductive tract. **a** Solidified ultra-LA dolutegravir surgically removed 7 days after administration. Scale bar represents 1 cm. **b** Pharmacological profile of NSG and BLT mice administered ultra-LA dolutegravir (250 mg/kg), five independent experiments, *n* = 21. **c** Dolutegravir concentrations in plasma and parts of female reproductive tract 7, 28, and 84 days after subcutaneous administration of ultra-LA dolutegravir (250 mg/kg). The data from two independent experiments are shown, *n* = 6 for each time point. Shown are individual animals and the means ± s.e.m. (standard error of the mean). Tissue density of 1 g/ml was used to compare dolutegravir concentration between tissues and plasma. **d** Plasma concentration of dolutegravir in 2 rhesus macaques administered 1 ml of formulation used in (**b**) and **c** (100 mg of dolutegravir)

Dolutegravir, a highly effective second-generation HIV integrase strand transfer inhibitor with extensive track record of efficacy and safety^15^, was used for preparation of ultra-LA formulation. In addition to active drug, the formulation consists of two relatively low cost FDA-approved excipients: (1) poly(lactic-*co*-glycolic acid) (PLGA), a biodegradable copolymer that eventually and safely biodegrades and, (2) *N*-methyl-2-pyrrolidone (NMP), a water miscible and biocompatible organic solvent^16,17^. Composition of the ultra-LA formulation was first optimized for release kinetics in vitro and then sustained delivery of dolutegravir in vivo (Fig. 1). The formulation contained dolutegravir/PLGA/NMP at a ratio 0.3:1:2 by weight, had a viscosity 845 cP at 25 °C (Brookfield Cone and Plate Digital Rheometer, *n* = 3). Formulation stability was assessed as the ability to remain in solution (measured by dynamic light scattering, Zetasizer Nano ZS Particle Analyzer), and to maintain a stable dolutegravir concentration (98% of the original concentration) measured by HPLC analysis. Dolutegravir was chemically stable in this formulation at 25 °C for at least 6 months. In the aqueous environment in vitro (0.01 M PBS, pH = 7.4, 2% solutol) solidification of the implant was instantaneous.

### Pharmacokinetics of ultra-LA dolutegravir

For an initial in vivo evaluation, anesthetized female NSG and BLT mice received a single subcutaneous injection of the ultra-LA dolutegravir on their back (5.5–7.0 mg dolutegravir in 50–80 µl). The injected formulation first formed a hard translucent globule under the skin that turned yellowish white (within 48 h of administration) as the formulation solidified. The formulation was well tolerated by the mice and no injection site reactions or other signs of overt toxicity, changes in behavior, movement, water consumption or weight loss were noted. For the purpose of observing the nature of the implant and to confirm that it could be readily removed from the mice, 1 week after administration a small incision was made near the location of the implant in one of the mice allowing rapid removal of the implant from the mouse (Fig. 1a). The rest of the mice were used for in vivo pharmacokinetic analysis of drug release (Fig. 1b). Plasma concentrations of dolutegravir were quantitated using a validated high-performance liquid chromatography–tandem mass spectrometry LC/MS–MS method^18^. Non-compartmental analysis of the median composite pharmacokinetic (PK) profile demonstrated a biexponential decay. After an initial 1st order decline in plasma concentrations, the release of dolutegravir approached zero-order kinetics. Plasma concentration of dolutegravir was ten times greater than the protein adjusted (PA)-IC_90_ for at least 5 months post administration. Even at 283 days after ultra-LA dolutegravir administration 1/3 mice still had detectable dolutegravir in plasma. We then used a sparse PK analysis to compare the concentration of plasma dolutegravir between BLT humanized and non-humanized mice. Our results demonstrated similar pharmacokinetics of dolutegravir in both types of mice (Supplementary Fig. 1).

Concentration of dolutegravir was also evaluated in the female reproductive tract (FRT) in 14 NSG mice receiving a single subcutaneous injection of ultra-LA dolutegravir. Vagina, cervix, uterus, and plasma from treated mice were collected 1, 4, and 12 weeks post administration and dolutegravir concentrations determined (Fig. 1c). One week after ultra-LA dolutegravir administration, the median concentration of dolutegravir in plasma, vagina, cervix, and uterus were 1350 ng/ml, 196 ng/mg, 158 ng/mg, and 272 ng/ml, respectively. One month post administration, the median dolutegravir concentrations were 958 ng/ml, 233 ng/mg, 262 ng/mg, and 303, ng/mg, respectively, and 12 weeks post administration the median concentrations were 1200 ng/ml, 356 ng/mg, 170 ng/mg, and 284 ng/mg, respectively (Fig. 1c, Table 1). Differences in DTG concentrations within each compartment (vagina, cervix, uterus, and plasma) comparing 1 week, 4 weeks, and 12 weeks (*n* = 6 per group) did not reach statistical significance (Kruskal–Wallis test plasma *p* = 0.21, cervix *p* = 0.09, uterus *p* = 0.70, vagina *p* = 0.17). Observations were combined over weeks 1, 4, and 12 to evaluate whether plasma concentrations were higher than tissue concentrations for each tissue type separately. Dolutegravir concentrations in plasma were higher than in tissue for each of the three tissue types for every animal (Wilcoxon signed-rank *p* < 0.001 for cervix, uterus, and vagina analyses, respectively). Together, these results demonstrate the sustained in vivo release of dolutegravir into plasma and its efficient penetration into tissues of the female reproductive tract.

**Table 1:** Tissue/plasma ratio of dolutegravir in female reproductive tract

	Day 7	Day 28	Day 84
Cervix	0.136	0.277	0.184
Uterus	0.191	0.345	0.208
Vagina	0.160	0.354	0.252
Mean ± SD	0.162 ± 0.03	0.326 ± 0.04	0.215 ± 0.03

Female NSG mice (*n* = 18) received 250 mg/ultra-LA dolutegravir subcutaneously and dolutegravir concentration in plasma and isolated organs of the female reproductive tract was analyzed at day 7 (*n* = 6), 28 (*n* = 6), and 84 (*n* = 6) using a LC–MS/MS method. Mean of ratios tissue/plasma is shown

To assess safety and drug release profile of the ultra-long-acting formulation of dolutegravir in a large animal model, two rhesus macaques were subcutaneously administered the ultra-LA dolutegravir formulation (100 mg). Animals were monitored for signs of toxicity and skin reactions at the injection site, including erythema, edema, and hematoma formation, presence of induration, and any other lesions such as abscesses, necrosis, dehiscence, or local inflammation twice a week. The implants were well tolerated with little or no sign of toxicity for 5 months (last point analyzed). Administration of the ultra-long-acting formulation of dolutegravir resulted in sustained dolutegravir concentration in plasma for more than 140 days (Fig. 1d). These results demonstrate the feasibility of ultra-long-acting dolutegravir delivery system for sustained delivery in rhesus macaques. However, this formulation originally developed for BLT mice will have to be further optimized prior to efficacy studies in macaques and its potential future applications in humans.

### Inhibition of HIV-1 replication ex vivo

**Fig. 2:**
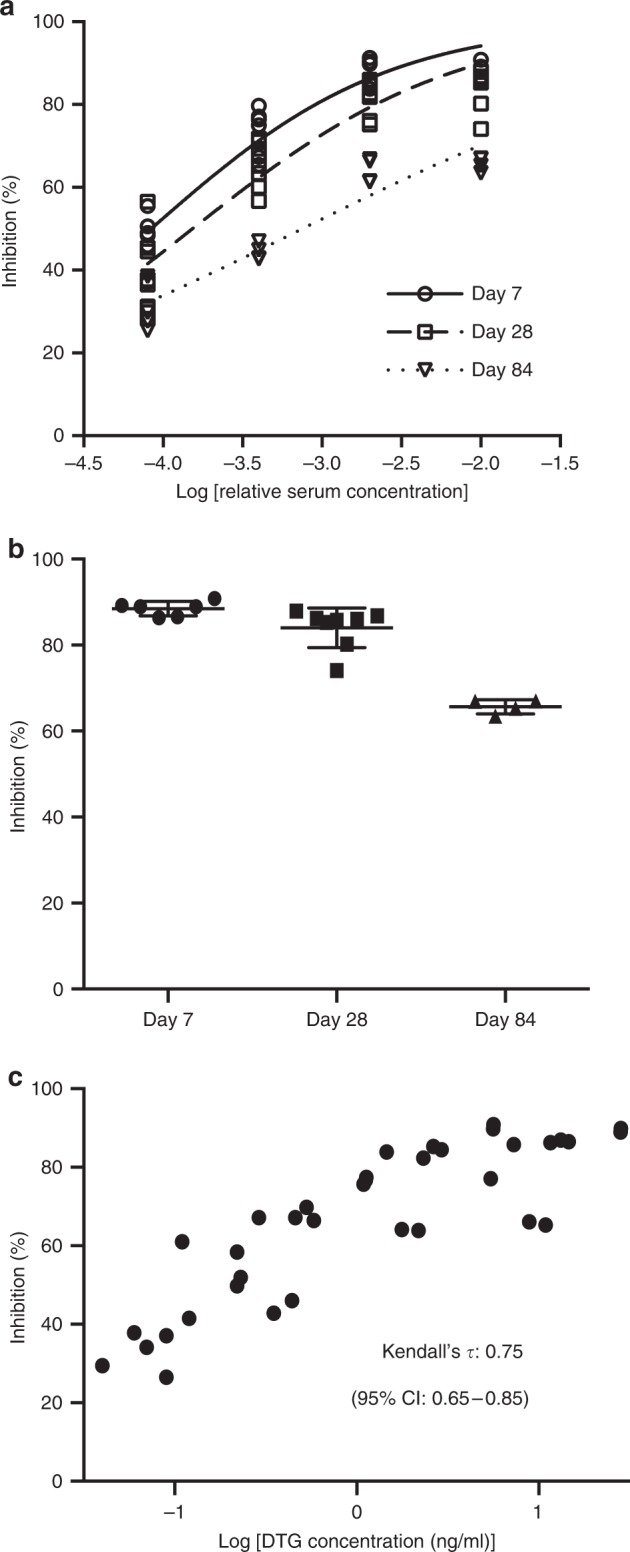
In vitro inhibition of HIV-1 infection with serum from ultra-LA dolutegravir-treated mice. Serum from female NSG mice administered with ultra-LA dolutegravir (250 mg/kg) collected at days 7 (*n* = 3), 28 (*n* = 4), and 84 (*n* = 2) was used for a TZM-bl cell-based assay (measured in duplicates). **a** Inhibition of HIV-1 infection (%) with various dilution of serum. Solid lines indicate nonlinear curve fit for the data. **b** Comparison of in vitro inhibitory activity of 1% serum collected from ultra-LA dolutegravir-treated NSG mice at the indicated time points (means ± s.e.m). **c** Comparison of in vitro inhibitory activity (%) of serum from ultra-LA dolutegravir-treated mice and log_10_ concentration of dolutegravir. A non-parametric rank-based correlation analysis accounting for clustered observations (Kendall’s tau) was used

Having demonstrated sustained concentration of dolutegravir in plasma of both mice and macaques and in tissues from mice we proceeded to evaluate its antiviral activity. Serum obtained from the ultra-LA dolutegravir-treated mice in Fig. 1c demonstrated strong, concentration dependent antiviral activity (Fig. 2a). Specifically, a one hundredth-fold dilution of serum was able to block in vitro viral infection by >86% when collected 7 or 28 days post administration (median 89%, range 86.4%–90.8% and median 86%, range 74.1%–87.9% for 7 and 28 days, respectively). A 100-fold dilution of serum was also able to block HIV infection by 66% when collected 84 days post ultra-LA dolutegravir administration (range 63.5%–67.0%) (Fig. 2b). Statistical analysis of the antiviral activity present in serum demonstrated a strong correlation with dolutegravir concentrations (Kendall rank correlation coefficient 0.75; 95% CI: 0.65–0.85) (Fig. 2c).

### Inhibition of HIV-1 replication in vivo

**Fig. 3:**
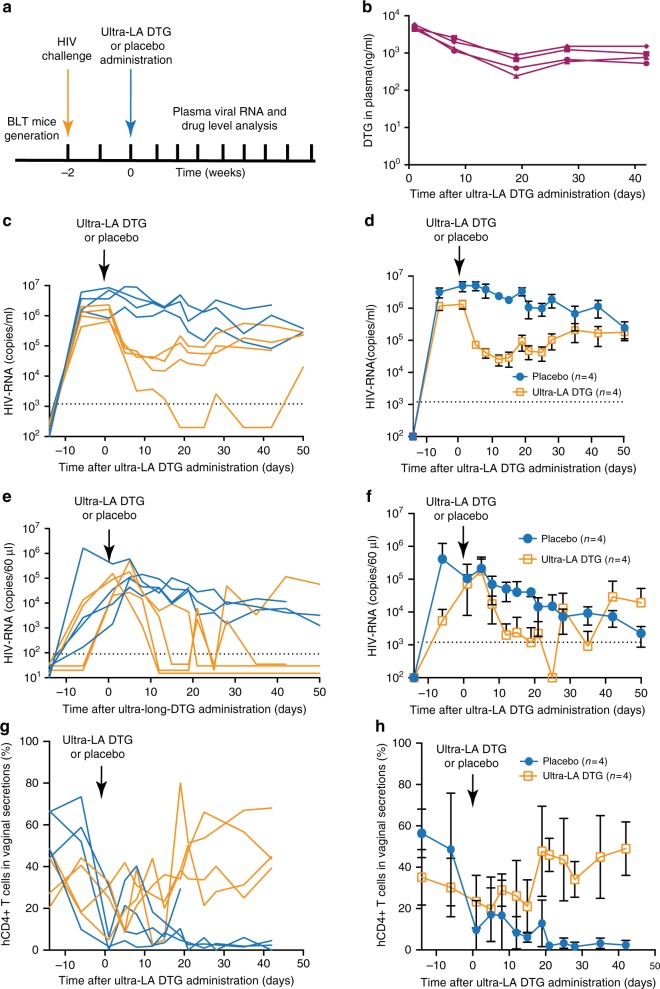
Suppression of systemic HIV infection by ultra-LA dolutegravir. **a** Experimental design. BLT mice infected with HIV-1_JRC-SF_ were subcutaneously administered ultra-LA dolutegravir (*n* = 4) or placebo (*n* = 4) and monitored for plasma dolutegravir and HIV-RNA concentrations. **b** Concentration of dolutegravir in plasma from ultra-LA dolutegravir-treated mice. Plasma HIV-RNA concentration in individual mice **c** or means ± s.e.m. **d** HIV-RNA in cervico-vaginal secretions (CVS) of individual mice (**e**) or means ± s.e.m. **f** CD4^+^ T cells (relative amount of CD3^+^ cells) in CVS of individual mice (**g**) or means ± s.e.m. **h** Yellow lines in **c**, **e**, and **g** ultra-long-acting dolutegravir-treated mice, blue lines placebo-treated mice. Experiment was conducted once

To establish the in vivo inhibitory effect of dolutegravir administered via the ultra-LA dolutegravir formulation on HIV replication, eight BLT humanized mice were first infected intravenously with HIV-1_JR-CSF_ (3 × 10^4^ TCIU) (Fig. 3a, Table 2). HIV-RNA was readily detected in plasma from all exposed mice 2 weeks after exposure (median 1.97 × 10^6^ copies/ml, range 0.65–8.50 × 10^6^ copies/ml). Two weeks post infection, mice received a single dose of ultra-LA dolutegravir- or placebo administered via subcutaneous injection (*n* = 4 for each group). Dolutegravir plasma concentrations in treated mice were sustained throughout the entire experiment (Fig. 3b). Placebo-treated mice maintained high concentration of plasma HIV-RNA. In contrast, strong suppression of HIV replication (~1–2 log) was noted in all mice treated with the ultra-LA dolutegravir formulation (Fig. 3c, d). In one mouse, viral load fell below the level of quantitation (LOQ, 1375 copies of HIV-RNA/ml) as early as 2 weeks post administration (Fig. 3c). Plasma viral load AUC was smaller in the ultra-long-acting dolutegravir group compared to the placebo group, *p* = 0.03 (exact Wilcoxon–Mann–Whitney test, *n* = 8). Virus RNA from plasma of mice treated with ultra-LA dolutegravir for 19 and 50 days were sequenced to determine whether mutations associated with drug resistance were acquired during this course of dolutegravir monotherapy. At day 19 bulk RNA sequences were obtained from the three mice with detectable viral loads. All three had a single nucleotide substitution resulting in an amino acid change at position 157 (E→K). At day 50, viral RNA from all four-treated mice was analyzed. At this time point, all three mice analyzed at day 19 had mutated from 157K to 157Q. In addition, in one mouse a mutation was also detected at position 263 (R→K). On day 50, the viral RNA from the mouse that was below detection at day 19 and thus could not be analyzed, had a mutation at position 157 (E→K). Sequencing of individual clones from all viral RNAs obtained at days 19 and 50 revealed the presence of several other mutations (Table 3). The majority of them were naturally occurring polymorphic substitutions. Interestingly, both the R263K and E157Q mutations were found in 2/8 clones from one mouse. Together, these two mutations have been shown to increase the resistance of HIV to dolutegravir in vitro^19^.

**Table 2:** HIV-1-RNA suppression by ultra-LA dolutegravir

Mouse	hCD45(%)	hCD4 (%)	Nadir (HIV-RNA copies/ml plasma)	Duration of suppression below LOQ in CVS (days)
S1	37.9	76.1	LOQ	54
S2	60.3	77.5	14,746.3	27
S3	43.6	71.5	38,282.0	16
S4	53	75.6	30,863.0	41
Mean	48.7	75.2	21,022	33.5
SD	9.9	2.56	17,000	15.0

BLT mice with indicated amount of human CD45^+^ (hCD45) cells and human CD3^+^CD4^+^ (hCD4) in peripheral blood were intravenously infected with HIV-1_JRC-SF_ and treated with ultra-LA dolutegravir formulation (250 mg/kg) 3 weeks later. Limit of quantitation (LOQ) was 1375 copies/ml in plasma and 81 copies/60 µl in CVS

CVS cervico-vaginal lavage, SD standard deviation

**Table 3:** DTG resistance development during ultra-LA-DTG monotherapy

Mouse	Dolutegravir exposure (days)	Identified mutations	Clones with the mutation/ Clones analyzed
S1	19	NA	NA
	50	E157K	5/5
S2	19	E157K	1/9
		E157K+Q35K+Q85K+R269G	1/9
		V260A	1/9
	50	R269K	1/9
		W243stop	1/9
		E157K	2/6
		E157Q	1/6
		**R263K**	1/6
S3	19	E157K	2/5
		K159N	1/5
		D3R+G4E	1/5
	50	E157Q	1/5
		E157Q+R166K+D41N+V281M	1/5
S4	19	D229G	1/9
	50	G70K	1/8
		D6E	1/8
		E157Q	1/8
		**R263K**	1/8
		**R263K**+E157Q	2/8

BLT humanized mice infected intravenously with HIV-1_JR-CSF_ (3 × 10^4^ TCIU) received a single dose of ultra-LA dolutegravir (*n* = 4) subcutaneously. Viruses in plasma at day 19 and 50 of the ultra-LA dolutegravir treatment were cloned and sequenced. Mutation previously described or identified as providing resistance to dolutegravir in bold

To determine the effect of ultra-LA dolutegravir on the concentration of HIV-RNA in the female reproductive tract mice were lavaged at the indicated time points and samples analyzed for the presence of HIV-RNA and dolutegravir concentrations. Within 2–3 weeks of treatment the concentration of HIV RNA in cervico-vaginal secretions (CVS) rapidly decreased below LOQ (81 HIV-RNA copies per 60 µl) with transient low-level viral increases in two mice (Fig. 3e, f). These results indicate that despite the observed lower concentration of dolutegravir in the FRT compared to plasma (Table 1), the dolutegravir concentration in FRT was sufficient to efficiently suppress viral replication (Table 2). A slow decrease in CVS HIV-RNA was also observed in the placebo-treated mice, most likely as a result of the dramatic reduction in CD4 T-cell numbers occurring during HIV infection in the FRT (Fig. 3h)^11^. In contrast to the dramatic depletion of CD4^+^ T cells noted in CVS from control (i.e., not treated) mice, in ultra-LA dolutegravir-treated mice, CD4 T-cell numbers progressively increased to the pre-exposure levels. CD4^+^ T-cell percentage AUC was larger for all mice in the ultra-long-acting dolutegravir group compared to the control group, *p* = 0.03 (exact Wilcoxon–Mann–Whitney test, *n* = 8) further demonstrating the efficacy of ultra-LA dolutegravir treatment on systemic HIV infection (Fig. 3g, h).

### Protection from vaginal HIV acquisition

**Fig. 4:**
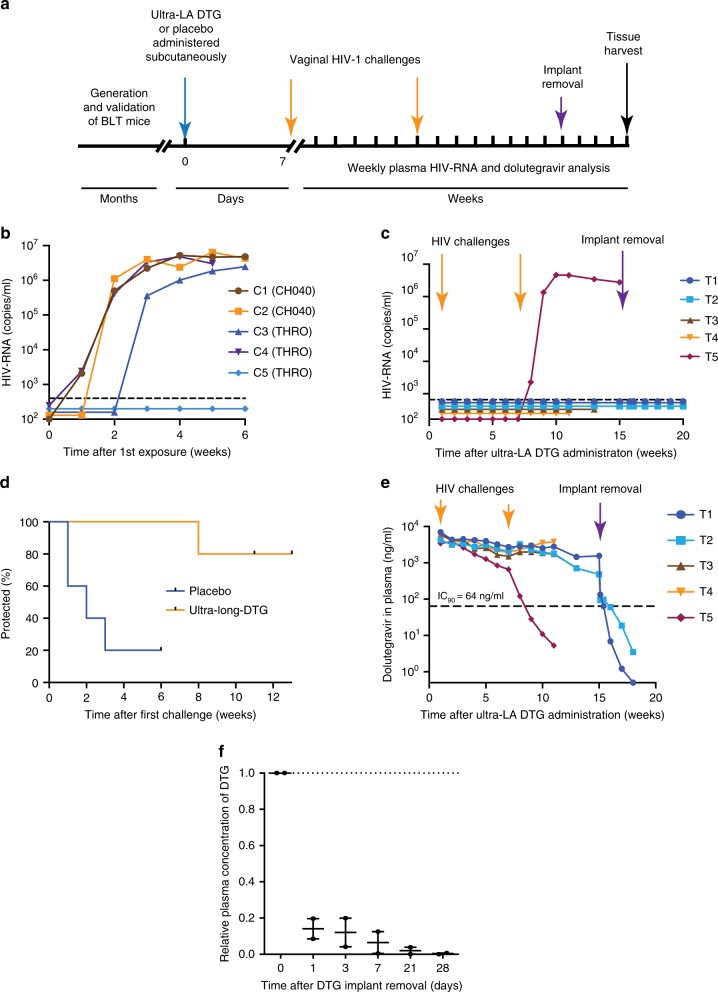
A single dose of ultra-LA dolutegravir protects against multiple high dose HIV-1 challenges. **a** Experimental design. BLT mice were treated with ultra-LA dolutegravir (*n* = 5) or placebo (*n* = 5) and vaginally exposed to HIV_CH040_ or HIV_THRO_ 1 week, and 7 weeks later. The implant was removed from two mice 8 weeks after second HIV challenge. Cell-associated HIV-DNA in multiple tissues was analyzed at the end of the experiment. Plasma HIV-RNA concentration in control (**b**) and treated mice (**c**). **d** Time-to-events plot illustrating the estimated probability of protection (*p* = 0.02). **e** Plasma concentration of dolutegravir. **f** Plasma concentration of dolutegravir (*n* = 2) after the implant removal relative to dolutegravir concentration immediately before the implant removal (dotted line). Individual measurements and median ± range is shown. Experiment was conducted once

To evaluate the potential of the ultra-LA dolutegravir to prevent vaginal HIV transmission, BLT mice (*n* = 5 per group) were subcutaneously administered ultra-LA dolutegravir (treated mice) or placebo (control mice) (Fig. 4a). Seven days later, mice were challenged vaginally with a high dose of one of two transmitted/founder viruses HIV-1_CH040_ (3.0 × 10^5^ TCIU, 2 controls, three treated mice) or HIV-1_THRO_ (3.5 × 10^5^, three controls, two treated mice). The two control mice exposed to HIV-1_CH040_ became plasma HIV-RNA positive within 2 weeks after challenge and 2/3 control mice challenged with HIV-1_THRO_ became plasma HIV-RNA positive within 3 weeks after the challenge (Fig. 4b). All ultra-LA dolutegravir-treated mice remained HIV negative after the first exposure. Six weeks after first challenge, ultra-LA dolutegravir-treated mice were challenged vaginally a second time with a high dose of HIV-1_CH040_ (3.0 × 10^5^ TCIU). One-treated mouse became HIV-RNA positive 1 week after the second challenge, and sequence analysis identified the breakthrough virus as HIV-1_CH040_ with no mutations in the HIV integrase gene. All other treated mice (4/5) remained negative for plasma HIV-RNA (Fig. 4d, Table 4). Two different but complementary approaches were used to exclude the possibility that HIV-1 was transmitted but suppressed due to the continuous presence of dolutegravir. First, two mice without evidence of HIV-RNA in plasma were collected 11 and 13 weeks post ultra-LA dolutegravir administration for tissue HIV-DNA analysis. No evidence of HIV DNA was noted in any of the tissues analyzed confirming systemic protection from infection. Second, the ultra-LA dolutegravir implant was surgically removed in the remaining two HIV-1-negative mice 15 weeks post-implantation. Implant removal resulted in a rapid decrease of plasma dolutegravir concentrations (>1 log within first 3 days after the removal) (Fig. 4f). After implant removal, no HIV-RNA was detected in plasma (Fig. 4c). Four weeks post-removal of the ultra-LA dolutegravir mice were killed, tissues were collected and analyzed for the presence of cell-associated HIV-DNA. No evidence of HIV-DNA was found in any of the tissues analyzed confirming that the lack of viremia after ultra-LA dolutegravir removal was not due to an occult infection masked by the continuous presence of high concentration of drug (Table 4). Rather these results strongly indicate that these four mice were fully protected from two high dose vaginal exposures to HIV 6 weeks apart.

**Table 4:** BLT mice protection with ultra-LA-DTG from high dose vaginal HIV-1 challenges

Mouse	hCD45 (%)	hCD4 (%)	Treatment	Virus for 1st challenge	Virus for 2nd challenge	Infecting virus	Presence of viral DNA in tissues					
							Lymph nodes	Spleen	Liver	Lung	Bone marrow	Org.
C1	49.6	85.9	Vehicle	CH040	None	CH040	+	+	+	+	+	+
C2	84.0	92.4	Vehicle	CH040	None	CH040	+	+	+	+	+	+
C3	74.0	86.9	Vehicle	THRO	None	THRO	+	+	+	+	+	+
C4	84.3	87.2	Vehicle	THRO	None	THRO	+	+	+	+	+	+
C5	94.3	82.0	Vehicle	THRO	None	THRO	+	+	+	+	+	+
T1	74.6	87.5	LA-DTG	CH040	CH040	None	−	−	−	−	−	−
T2	84.4	88.4	LA-DTG	CH040	CH040	None	−	−	−	−	−	−
T3	73.2	89.8	LA-DTG	THRO	CH040	None	−	−	−	−	−	−
T4	80.2	91.9	LA-DTG	THRO	CH040	None	−	−	−	−	−	−
T5	67.2	93.0	LA-DTG	CH040	CH040	CH040	+	+	+	+	+	+
Mean	76.6	88.5										
SD	21.0	12.2										

BLT mice with indicated amount of human CD45^+^ (hCD45) cells and human CD3^+^CD4^+^ (hCD4) were administered with ultra-long-acting dolutegravir formulation (250 mg/kg) or placebo subcutaneously. One week after treatment mice were vaginally exposed to the indicated HIV-1 isolate. Six weeks later mice were re-exposed to HIV-1_CH040_. DTG-dolutegravir. Cell-associated DNA was analyzed in indicated tissue

*−* negative for viral DNA, *+* positive for viral DNA, *org.* thymic organoid

In order to establish a possible cause for the single breakthrough infection noted above, plasma drug concentrations throughout the course of the experiment were analyzed. The concentration of dolutegravir in plasma of 4/5 treated mice was maintained at 2805 ng/ml (median, range 1510–4510 ng/ml) for 11 weeks. In the mouse with the breakthrough infection, plasma dolutegravir concentration began to slowly decrease 3 weeks after injection. At the time of the second viral challenge, the plasma dolutegravir concentration was 659 ng/ml. Plasma dolutegravir concentration continued to drop and by 11 weeks post ultra-LA dolutegravir administration it was below 10 ng/ml (Fig. 4e). These results suggest that the breakthrough infection is likely due to the lower concentrations of dolutegravir in this mouse.

## Discussion

The HIV epidemic continues to be a significant health concern worldwide. In 2016, ~36.7 million people were living with HIV^20^. Among promising preventive interventions is pre-exposure prophylaxis (PrEP), in which ARVs are taken by HIV-negative people before potential exposure to the virus. Clinical studies established that daily oral PrEP with Truvada^®^ can prevent HIV infection among high-risk populations^21–25^. However, recent clinical trials demonstrate that lack of adherence to the indicated drug regimen is common, resulting in lack of protection from HIV infection^26,27^.

In the specific case of HIV prevention, the lack of adherence by clinical trial participants has served to highlight the urgent need for drug delivery systems capable of offering long-term protection from HIV infection^26,27^. Two LA-ARV formulations for HIV PrEP are in clinical trials. Cabotegravir, an integrase strand transfer inhibitor (INSTI), is in phase IIa and IIb/III clinical trials^28^ and rilpivirine, a non-nucleoside reverse transcriptase inhibitor (NNRTI), is in a phase II clinical trial^29^. Both drugs are formulated as nanosuspensions for LA intramuscular injections every 8 weeks^5–7^. Major limitations of this type of drug formulations are the complete inability to remove the injected nanosuspension from the body in the case of a medical emergency and the inability of co-formulation of two or more drugs. Removal of LA formulations is essential to circumvent adverse reactions or to prevent long-term sub-therapeutic drug exposure after PrEP discontinuation. These concerns are currently being partially addressed by an oral regimen of drug for 5 weeks prior to the administration of the LA formulation and by daily oral administration of Truvada for up to 1 year at the end of a dosing regimen^7^. Given the documented evidence of lack of compliance with this approach, these options are likely not appropriate for the targeted populations.

Humanized mouse models were used for pre-clinical efficacy assessment of LA formulations of ARVs. A nanosuspension of crystalline rilpivirine, administered intramuscularly, protected BLT mice from a single vaginal high-dose HIV-1 challenge 1 week after drug administration and provide partial protection 4 weeks after drug administration^12^. Similarly, single subcutaneous dose of LA raltegravir significantly protected BLT mice from vaginal HIV acquisition 4 weeks after drug administration^13^. Recently, a lipophilic modified DTG prodrug encapsulated into poloxamer nanoformulations significantly protected CD34+ humanized mice from the HIV-1 for 2 weeks^30^. These results suggest utility of humanized mouse models for pre-clinical evaluation of LA formulation of AVR.

Optimally, a LA delivery system for HIV prophylaxis should be effective, safe, easy to apply, and affordable so that it can be used in resource-poor and constrained settings. The data in this paper indicate that ultra-LA dolutegravir can be readily prepared and sterilized by filtration. It can efficiently deliver dolutegravir for up to 9 months in mice and 140 days in NHP (last time point analyzed). A single administration of ultra-LA dolutegravir strongly inhibited acute HIV replication, and effectively protected against repeated high-dose vaginal HIV challenges using highly relevant primary transmitted/founder viruses. An important aspect of the ultra-LA dolutegravir formulation is the fact that it can be easily removed resulting in rapid decrease of drug concentration providing a measure of safety that is not afforded by any of the current LA drug delivery systems in clinical trials for HIV prevention. However, when immediate removal is not necessary, the formulation is biodegradable and does not require surgical removal. In addition, the formulations offer the flexibility to include multiple drugs and the future potential to be reloadable in situ^31^.

In this stage of formulation development, it is difficult to predict how much ultra-LA dolutegravir will be needed to achieve same efficacy of prevention from HIV transmission as seen in BLT mice. However, we can compare this formulation to existing LA formulations in clinical trials. Cabotegravir nanoformulation consists of two 2-ml injections of product containing 200 mg/ml drug administered every 2 months. Our goal is to develop a formulation that can accommodate 250 mg/ml dolutegravir. As the IC90 for dolutegravir (64 ng/ml) is 2.5-fold lower that the IC90 for cabotegravir (166 ng/ml) we consider this to be an appropriate target. PK analysis shown in Fig. 1 also suggests longer sustained release of dolutegravir in vivo compared to the current cabotegravir formulation. Therefore, using the same parameters currently used for cabotegravir, our ultra-LA dolutegravir is likely scalable to humans.

This technology was inspired from the Atrigel product that is commercially available as Eligard (please see citation 8 and 9). This is a palliative treatment for advanced prostate cancer. However, there are several differences between Eligard and our ultra-LA delivery system. (1) Eligard is packaged as a 2-syringe system due to the limited stability of leuprolide acetate in the PLGA/NMP formulation and administered as a dispersion rather than a solution^32^. (2) The ultra-LA dolutegravir formulation presented in this manuscript uses an antiretroviral drug instead of a synthetic hormone, (3) the formulation is prepared as a solution that is stable for more than 6 months at room temperature. Since the Atrigel technology has already been approved by the FDA for treatment of prostate cancer we anticipate that it is also applicable for HIV PrEP.

Several key observations are worth highlighting. Dolutegravir monotherapy resulted in strong and sustained suppression of HIV replication. However, as early as 19 days post therapy initiation, resistance mutations begun to appear in the replicating viruses. By day 50 post therapy initiation, both drug resistance and compensatory mutations were found. Consistent with studies in women^18^, the concentration of dolutegravir in FRT tissues from BLT mice was 3–7 times lower than in plasma. However, even under these conditions significant protection from high dose vaginal infection was noted. The one case of breakthrough infection could be attributed to the lower drug concentration present in this mouse at or near the time of exposure (659 ng/ml). Of note, the dolutegravir plasma concentrations sustained in the mice protected from high dose HIV infection (~2800 ng/ml) are directly comparable to those of cabotegravir reported to be protective in a non-human primate model of low dose repeated exposure vaginal SHIV infection^33^. In summary, the results demonstrate the in vivo effectiveness of an ultra-LA formulation of dolutegravir to deliver drug for up to 9 months that results in sustained viral suppression and prolonged protection from high dose HIV vaginal challenges. With its ultra-long duration, low cost of production, ease of administration and the ability to be removed this represents a significant advance in drug delivery for HIV pre-exposure prophylaxis.

## Methods

### Chemicals

50:50 Poly(DL-lactide-*co*-glycolide) was purchased from LACTEL (Birmingham, AL; Cat. No. B6010–1P, MW 27 kDa). *N*-methyl-2-pyrrolidone (NMP) was purchased from ASHLAND (Wilmington, DE, Product Code 851263, 100% NMP). Dolutegravir (DTG) was purchased from Selleckchem (Houston, TX; Cat. No. S2667, 99.8% DTG).

### Preparation of ultra-LA dolutegravir formulations

In a 7 mL scintillation vial back-filled with argon, 50:50 Poly(DL-lactide-*co*-glycolide) (PLGA) was mixed with *N*-methyl-2-pyrrolidone (NMP) at a 1:2 PLGA/NMP weight ratio (w/w) and allowed to dissolve by continuous mixing at room temperature (Placebo). Formulation was sterilized by filtration (0.2 µm filter). Dolutegravir (DTG) was subsequently added to the PLGA/NMP solution and stirred at 40 °C for 2 h to dissolve DTG and produce an ultra-LA dolutegravir formulation with a final concentration of dolutegravir 93 mg/mL (ultra-LA dolutegravir; DTG/PLGA/NMP = 0.3:1:2 w/w/w). The viscosity of the ultra-LA dolutegravir (845 cP) was measured using a Brookfield Cone and Plate Digital Rheometer (Model: LVDV-III+CP) and the reading was recorded at 25 °C and 1 rpm. The chemical stability of DTG in the ultra-LA dolutegravir formulation (DTG/PLGA/NMP 0.3:1:2 w/w/w) was measured at 25 °C over 90 days. The ultra-LA dolutegravir formulation remained as a solution for 90 days as measured by dynamic light scattering (DLS, a Zetasizer Nano ZS Particle Analyzer (Malvern Instruments, Inc.). In addition, DTG concentration at day 90 was ~98% the original concentration (91 ± 0.6 mg/mL) as measured by HPLC analysis using a Finnigan Surveyor HPLC system (Thermo Finnigan, San Jose, CA) with a Photodiode Array (PDA) Plus Detector, auto-sampler, and LC Pump Plus. The stationary phase utilized for the analysis was a Inertsil ODS-3 column (5 μm, 4.6 × 150 mm, [GL Sciences, Torrance, CA]) maintained at 40 °C. Chromatographic separation was achieved by gradient elution using a mobile phase consisting of 0.1% trifluoroacetic acid in water and acetonitrile (ACN) (H_2_O/ACN 95:5 v/v). The flow rate was 1.0 mL/min and the total run time was 25 min for each 25 μL injection. Implant formation in vitro was instantaneous upon injection of the formulation in the aqueous release medium (0.01 M PBS, pH = 7.4, 2% solutol).

### BLT humanized mice and non-human primates

BLT humanized mice were prepared by implanting a sandwich of human thymus-liver-thymus tissue under the kidney capsule of irradiated (200 rads) 8–12 weeks old female NOD/SCID/γc^−/−^ (NSG) mice (The Jackson Laboratory, Bar Harbor, ME). Following tissue implantation, mice received autologous CD34^+^ hematopoietic stem cells via tail vein injection. Starting at 8 weeks post transplantation, human immune cell reconstitution was monitored in the peripheral blood of BLT mice by flow cytometry longitudinally. Specifically, concentration of human cells (human CD45+ cells, BD Pharmingen™ APC Mouse Anti-Human CD45, cat. #555485, 1:100), human T cells (human CD45+CD3+ cells, BD Pharmingen™ FITC Mouse Anti-Human CD3, cat. #555339, 1:100) and human CD4 T cells (human CD45+CD3+CD4+ cells, BD Pharmingen™ PE Mouse Anti-Human CD4, cat. #555347, 1:100) were determined and mice with ≥40% human CD45^+^ cells (~3 months after humanization) were used for experiments^12,34–37^. Due to the focus on prevention of vaginal HIV transmission, only female mice were used for the experiments. Mice were maintained by the Division of Comparative Medicine at UNC-Chapel Hill according to protocol approved by the Institutional Use and Care Committee under AAALAC guidelines. Two rhesus macaques (*Macaca mulatta*) were used for the in vivo evaluation of the ultra-LA dolutegravir. Animals were maintained at the CDC Roybal Campus animal facility in Atlanta, Georgia, all procedures were performed by the Comparative Medicine Branch (CMB) with certified attending DVMs and trained staff. All animal protocols have been approved by the onsite IACUC under AAALAC guidelines.

### Administration of ultra-LA-DTG and sample collection

Liquid ultra-LA dolutegravir (250 mg/kg in 50–80 µl) or a similar volume of placebo formulations were administered subcutaneously with a 19 G needle on the shaved back of anesthetized BLT or NSG mice. Peripheral blood was collected from mice into capillary tubes coated with or without EDTA to isolate plasma or serum, respectively. CVS were obtained by lavage with sterile PBS (three washes of 20 μl each, ~60 μl total volume). All samples were stored at −80 °C until analysis. Similarly, ultra-LA dolutegravir formulation was administered to two rhesus macaques via subcutaneous injection. The administration site was clipped of hair and aseptically treated with povidone iodine or chlorhexiderm and isopropanol on the day of implantation. Formulation was administered subcutaneously under anesthesia using a 19G needle. Injections were done above the thoracic vertebrae T1/T2^16^. We used a custom-made primate jacket and a T-shirt under to avoid animal scratching. Animals went through an acclimation period for the jackets prior to the implantation. The jackets are not restrictive and do not interfere with movement or eating. Peripheral blood samples were collected and analyzed for drug concentrations essentially as indicated above.

### Implant removal from BLT mice

Mice were anesthetized and their back was shaved to visualize the location of the implant. Implant was removed under sterile conditions via a small cutaneous incision adjacent to the implant and removing the implant using forceps.

### Analysis of dolutegravir in plasma and tissues

Quantification of DTG plasma concentrations was performed by protein precipitation and LC–MS/MS analysis. Calibration curves were obtained using a 1/concentration^2^ weighted linear regression of analyte:internal standard peak area ratio versus nominal concentration. Compilation of concentration results and descriptive statistical analyses were performed using Sciex Analyst version 1.6.1. Ten microliters of each stored plasma sample was mixed with 30 μL of acetonitrile containing the isotopically labeled internal standard, dolutegravir-d_7_^15^N (DTG-IS). Following vigorous mixing and centrifugation, a portion of the supernatant was diluted with 50:50 methanol:water prior to LC–MS/MS analysis. DTG was eluted from a Varian (Agilent) Pursuit Diphenyl (2.1 × 50 mm, 3 μm particle size) analytical column. An API- 5000 triple quadrupole mass spectrometer (AB Sciex, Foster City, CA) was used to detect the analyte. For DTG, the precursor ion was 420 m/z and the product ion was 277 m/z. For DTG-IS, the precursor ion was 428 m/z and the product ion was 283 m/z. The data were collected using AB Sciex Analyst Chromatography Software. The dynamic range of the assay was 1–10,000 ng/mL. All calibrators and quality control (QC) samples were within 15% of the nominal value for both within-day and between-day runs. The high, medium, and low QC values used for plasma were 3.00, 300, and 3000 ng/mL. Within-day and between-day precision calculations were <15%. The recovery range for DTG in plasma was 98.0–103%, and the recovery of DTG-IS was 104%.

Thirty microliters of each vaginal wash sample was mixed with 270 μL of acetonitrile containing DTG-IS. Following vortex and centrifugation, a portion of the supernatant was diluted with 50:50 methanol:water. DTG was detected using identical conditions to those described for plasma. The dynamic range of the assay was 1–1000 ng/mL. The recovery range for DTG in CVF was 99.4–105%, and the recovery of DTG-IS was 101%. All calibrators and quality controls samples were within 15% of the nominal value. The low, medium, and high QC values used for CVF were 3.00, 30.0, and 800 ng/mL. Within-day and between-day precision was <15%.

In order to extract DTG from tissue samples, they were initially homogenized in 1 mL of 80:20 water:acetonitrile. Fifty microliters of the resulting homogenate was extracted by protein precipitation with acetonitrile containing DTG-IS. Following vortex and centrifugation, a portion of the supernatant was diluted with water. DTG was detected on an LC–MS/MS system using identical conditions as described above. During method validation, calibration standards were prepared in mouse tissue homogenate. QC samples were prepared in mouse genital tract tissue homogenates. The dynamic range of the assay was 0.2–200 ng/mL homogenate. The recovery range of DTG in tissue homogenate was 50.7–63.4%, and the recovery of DTG-IS was 72.0%. All calibrators and quality controls samples were within 15% of the nominal value with precision values <15%. The low, medium, and high QC values used for tissues were 0.600, 6.00, and 160 ng/mL, respectively.

### Preparation of HIV-1 stocks for infection

Viral stocks of HIV-1_JR-CSF_, HIV-1_CH040_, and HIV-1_THRO_ were generated by transfecting proviral DNA^38–42^ into 293T cells (American Tissue Culture Collection, catalog number CRL-3216, passage <10) using Lipofectamine 2000 (Invitrogen). Viral particles were collected from tissue culture medium, pre-cleared by centrifugation (3000 RPM for 20 min at 4 °C) and concentrated by ultracentrifugation (31,000 RPM for 70 min at 4 °C). Tissue culture infectious units (TCID)/ml of HIV was determined by titration on TZM-bl cells (NIH AIDS Research and Reference Reagent Program, catalog number 8129, passage number 2–6), infected TZM-bl cells were visualized by with staining solution (4 µM potassium ferrocyanide, 4 µM potassium ferricyanide, 2 µM magnesium chloride, 0.4 mg/ml X-gal)^12,43^. TZM-bl and 293T cells were cultured at 37 °C, 10% CO_2_ in Dulbecco’s Modified Eagle Medium (Sigma) supplemented with 10% fetal bovine serum, 25 mM HEPES, 500 units/ml penicillin, 50 µg/ml streptomycin and 2 mM l-glutamine (Cellgro), cells were regularly checked for morphology by microscope.

### In vitro HIV-1 inhibition

TZM-bl cells were maintained in TZM-bl medium as described above and cultured at 37 °C and 5% CO_2_. TZM-bl cells were plated in 96-well plates at a density of 1 × 10^5^ cells per well in TZM-bl medium. The next day, the medium was removed and 100 μl of diluted serum from dolutegravir-treated or placebo-treated mice collected 7 day, 28 days, or 84 days post administration was added. Serum was diluted 1:20, 1:100, 1:500, 1:2500, 1:12,500, and 1:62,500 and incubated with cells for 30 min. The cells were infected with 100 μl of TZM-bl medium containing 40 µg/ml of DEAE-dextran and 3 × 10^3^ TCIU of HIV-1_JR-CSF_ per well was added. Approximately 48 h later, the medium was removed and the luciferase substrate One-Glo reagent (Promega, Madison, WI) supplemented with 0.2% Triton X-100 was added to allow for the measurement of luciferase activity and to inactivate virus. Luciferase was measured with a SpectraMax M3 Spectrometer (Molecular Devices, Sunnyvale, CA) and the results normalized to the luciferase activity of cells infected with HIV in the absence of serum and expressed as a percentage of decrease in luciferase activity. The TZM-bl cells were obtained through the NIH AIDS Reagent Program (catalog number 8129, passage number 2–6), cells were regularly checked for morphology by microscope.

### HIV exposure of BLT mice

For the analysis of the effect of dolutegravir monotherapy on HIV replication, BLT mice were exposed intravenously to 3 × 10^4^ TCIU of HIV-1_JRC-SF_. Two weeks after infection mice were subcutaneously administered the ultra-LA dolutegravir (or placebo) formulation. To evaluate the ability of the ultra-LA dolutegravir (or placebo) preparation to prevent vaginal HIV transmission following multiple virus challenges, treated BLT mice were exposed vaginally to HIV-1_CHO40_ or HIV-1_THRO_ 1 and 7 weeks after ultra-LA dolutegravir administration. Anesthetized BLT mice were exposed vaginally to 3.0 × 10^5^ TCIU of HIV by pipetting virus (20 µl) directly into the vaginal cavity.

### Analysis of HIV infection

HIV infection was monitored longitudinally in peripheral blood of BLT mice by determining HIV-RNA concentration in plasma by one-step real-time reverse transcriptase PCR assay, using the following primers: CATGTTTTCAGCATTATCAGAAGGA, TGCTTGATGTCCCCCCACT, and the MGB-probe carboxyfluorescein (FAM)-CCACCCCACAAGATTTAAACACCATGCTAA-Q (non-fluorescent quencher) (Applied Biosystems) (assay sensitivity of 1375 HIV-RNA copies/ml). The presence of HIV-DNA in tissues (spleen, lymph nodes, bone marrow, human thymus, liver, lung, and FRT) collected from BLT mice at necropsy was determined by real-time PCR analysis of DNA extracted from isolated mononuclear cells (assay limit of detection: 28 copies) with same primers and HIV-RNA (Supplementary Table 1). As a control for the presence of DNA extracted from human cells, all samples were tested for the presence of human gamma globin DNA by real-time PCR using primers: CGCTTCTGGAACGTCTGAGATT (forward), CCTTGTCCTCCTCCTCTGTGAAATGA (reverse) (Supplementary table 1). Concentration of human cells (human CD45+ cells, BD Pharmingen™ APC Mouse Anti-Human CD45, cat. #555485, 1:100), human T cells (human CD45+CD3+ cells, BD Pharmingen™ FITC Mouse Anti-Human CD3, cat. #555339, 1:100) and human CD4 T cells (human CD45+CD3+CD4+ cells, BD Pharmingen™ PE Mouse Anti-Human CD4, cat. #555347, 1:100) were determined using a BD FACSCanto^TM^ flow cytometer and analyzed using FlowJo software (FLOWJO, LLC, version 10.0.8).

### Sequence analysis of infecting viruses

The identity of the infecting viruses was determined by sequence analysis. Viral RNA was isolated from plasma using QIAamp viral RNA columns (Qiagen, Hilden, Germany) according to the manufacturer’s protocol, and cDNA was generated using Superscript III Reverse Transcriptase (Invitrogen, Carlsbad, CA) with the primer 5′-CTTCCAATTATGTTGACAGGTGTAGG-3′. cDNA was amplified by nested PCR using the Expand High Fidelity PCR System (Roche, Mannheim, Germany). PCR primers were designed to anneal in regions with the fewest possible primer mismatches to HIV-1_CH040_, HIV-1_JR-CSF_, and HIV-1_THRO_ sequences. Primer sequences were as follows: outer forward primer, 5′-CTCAATAAAGCTTGCCTTGAGTGC-3′; outer reverse prime 5′-CTTCCAATTATGTTGACAGGTGTAGG-3′; inner forward primer, 5′-GTGTGGAAAATCTCTAGCAGTGGC-3′; inner reverse primer 5′-CTGTATCATCTGCTCCTGTATCTAATAGAGC-3′ (Supplementary Table 1). Amplified viral DNA of *gag* was sequenced and compared to the sequences of challenge viruses.

To identify the emergence of drug-resistant mutations in the breakthrough viruses from BLT mice receiving ultra-LA dolutegravir PrEP or BLT mice suppressed with ultra-LA dolutegravir monotherapy, viral RNA was extracted from plasma using QIAamp viral RNA kit (Qiagen, Hilden, Germany). cDNA was generated from RNA with Superscript III RT (Invitrogen, Carlsbad, CA) with primer 5′-GGTCAGGGTCTACTTGTGTGC-3′ followed by nested PCR with Expand High Fidelity Kit (Roche, Mannheim, Germany) to amplify 1.5 kb region of reverse transcriptase. The following primers were used: forward outer primer 5′-CCTGAGTGGGAGTTTGTCAATAC-3′, reverse outer primer 5′-GGTCAGGGTCTACTTGTGTGC-3′, forward inner primer 5′-AGCACACAAAGGAATTGGAG-3′ reverse inner primer 5′-GTGGGATTTGTACTTCTGAAC-3′ (Supplementary Table 1). Amplified viral DNA of integrase was sequenced and compared to the sequence of the original HIV-1 used for infection. To further characterize the mutations found, cDNA were cloned (6–10 clones), sequenced and analyzed. FinchTV software (Geospiza, Seattle, WA) was used to analyze sequence chromatograms, NCBI BLAST to identify sequence, and ClustalW to align sequences.

### Statistical analysis

All data were graphed using GraphPad Prism (version 6.0). The statistical tests used are indicated in the figure legends and/or text.

For PK analysis in plasma and tissues, dolutegravir concentrations (ng/ml or ng/g) in plasma, and tissue from the cervix, uterus, and vagina, (Fig. 1c) were measured in 18 mice with six mice observed at each time point (days 7, 28, and 84). Different mice contributed to each time point because the animals were collected to measure tissue concentrations; the same design was used for plasma analysis. Dolutegravir levels were compared between the three time points using an exact Kruskal–Wallis test to test for any statistically significant difference, with each sample type (plasma, cervix, uterus, vagina) analyzed separately. Ratios of plasma: tissue concentration within mouse were evaluated using an exact Wilcoxon signed-rank test [Ho: plasma: tissue ratio = 1, i.e., log(plasma DTG)–log(tissue DTG) = 0]; observations were combined over the three time points and each tissue type was analyzed separately. A 0.05 statistical significance level was used with no adjustment for multiple testing; two-sided tests were used throughout. Seven (7) null hypothesis significance tests were conducted in this report.

A non-parametric rank-based correlation analysis accounting for clustered observations (Kendall’s tau) was used to compare in vitro inhibitory activity (%) of serum from ultra-LA dolutegravir-treated mice and log_10_ concentration of dolutegravir (Fig. 2c)^44^.

For the suppression experiment (Fig. 3) the outcomes of plasma viral load, (CVS) cervical vaginal secretion viral load, and hCD4^+^ T-cell concentration, area under the curve (AUC) was calculated using the trapezoid method over 11 longitudinal measurements (taken between days 1 and 42) and compared between the control and dolutegravir-treated groups. A generalization of the exact Wilcoxon–Mann–Whitney test was used to account for interval-censored observations arising from HIV RNA results below the limit of detection (LOQ)^45^. Lower and upper bounds for the AUC were computed using zero and the LOQ, respectively, for results below the limit. In the absence of censoring (e.g., hCD4%) this test reduces to the exact Wilcoxon rank-sum test. Notably, one mouse in the control group died after six longitudinal measurements and was assigned the worst ranked AUC for analysis (i.e., the largest viral load AUCs and lowest hCD4 % AUC). Analyses were conducted in SAS version 9.4 (Cary, NC) and R-project version 3.4.0.

An exact log rank test was used to compare protection between placebo and ultra-LA dolutegravir-treated BLT mice (Fig. 4d). If not otherwise specified, standard error of the mean was used to estimate variation within each group. Variance is similar between the groups that are being statistically compared. No statistical methods were used to pre-determine the sample size. No randomization was used to determine how samples/animals were allocated to experimental groups. Mice representing the same human donor were allocated to each experimental group. Each experimental group had animals with similar levels of humanization. Blinding was used to analyze plasma concentration of HIV-RNA and concentration of cell-associated HIV-RNA and HIV-DNA. Investigators were given numbered samples, but not the allocation of the samples to groups (treated, controls). No other blinding was used.

### Code availability

Following commercially available software were used for data collection and analysis. For data collection (FACS), BD FACSDiva software (version 6.1.3) was used. The data were analyzed using FlowJo software (version 10), Microsoft Excel (2016), GraphPad Prism (versions 6), SAS version 9.4 and R-project version 3.4.0.

## Electronic supplementary material

Supplementary Information

## Data Availability

Sequence data that support the findings in Table 3 of this study have been deposited in GenBank with the accession codes: MH425196-MH425242. The authors declare that all other data supporting the findings of this study are available within the article and its Supplementary Information files, or are available from the authors upon request.
