# Supplementary material for: Ultra-long-acting removable drug delivery system for HIV treatment and prevention
Source: Nat Commun. 2018 Oct 8;9:4156. doi: 10.1038/s41467-018-06490-w (PMC6175887; doi:10.1038/s41467-018-06490-w)
Supplement: Supplementary file 1 — Supplementary Information [file 41467_2018_6490_MOESM1_ESM.pdf]

Supplementary information

Ultra-long-acting removable drug delivery system for HIV treatment and prevention

Kovarova et al

Supplementary Materials:

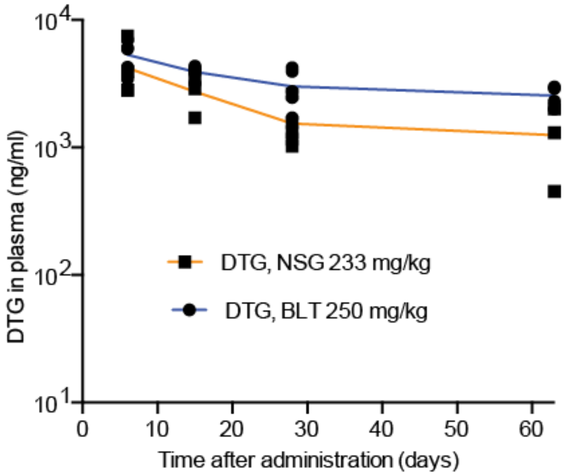

Supplementary Figure 1: Comparison of pharmacological profiles of ultra-LA DTG in NSG and BLT mice. NSG (n=3) or BLT (n=5) mice received subcutaneously 233 mg/kg and 250 mg/kg of ultra-long-acting dolutegravir, respectively. Plasma dolutegravir concentration was evaluated longitudinally for 2 months after ultra-long-acting dolutegravir administration. Concentration of DTG in plasma of individual mice are shown. Experiment was conducted once.

Supplementary Table 2: Sequences of primers used in the study

Use of the primer	Sequence of primer
Generation of HIV *gag* cDNA	5'-CTTCCAATTATGTTGACAGGTGTAGG-3'
Nested PCR of *gag;* outer forward primer	5'-CTCAATAAAGCTTGCCTTGAGTGC-3';
Nested PCR of *gag;* outer reverse prime	5'-CTTCCAATTATGTTGACAGGTGTAGG-3';
Nested PCR of *gag;* inner forward primer	5'-GTGTGGAAAATCTCTAGCAGTGGC-3';
Nested PCR of *gag;* inner reverse primer	5'-CTGTATCATCTGCTCCTGTATCTAATAGAGC-3'
Generation of HIV integrase cDNA	5'- GGTCAGGGTCTACTTGTGTGC -3'
Nested PCR of integrase; outer forward primer	5'- CCTGAGTGGGAGTTTGTCAATAC -3'
Nested PCR of integrase; outer reverse prime	5'- GGTCAGGGTCTACTTGTGTGC -3'
Nested PCR of integrase; inner forward primer	5'- AGCACACAAAGGAATTGGAG -3'
Nested PCR of integrase; inner reverse primer	5'- GTGGGATTTGTACTTCTGAAC -3'
Forward primer for HIV-RNA and DNA amplification	5'- CATGTTTTCAGCATTATCAGAAGGA-3'
Reverse primer for HIV-RNA and DNA amplification	5'- TGCTTGATGTCCCCCCACT-3'
Forward primer for human gamma globin DNA amplification	5'- CGCTTCTGGAACGTCTGAGATT-3'
Reverse primer for human gamma globin DNA amplification	5'- CCTTGTCCTCCTCCTCTGTGAAATGA -3'
